# Monitoring CSF Proteome Alterations in Amyotrophic Lateral Sclerosis: Obstacles and Perspectives in Translating a Novel Marker Panel to the Clinic

**DOI:** 10.1371/journal.pone.0044401

**Published:** 2012-09-06

**Authors:** Nils von Neuhoff, Tonio Oumeraci, Thomas Wolf, Katja Kollewe, Peter Bewerunge, Boris Neumann, Benedikt Brors, Johannes Bufler, Ulrich Wurster, Brigitte Schlegelberger, Reinhard Dengler, Marc Zapatka, Susanne Petri

**Affiliations:** 1 Institute of Cell and Molecular Pathology, Hannover Medical School, Hannover, Germany; 2 Computational Oncology, Division of Theoretical Bioinformatics, Research Program Structural and Functional Genomics, German Cancer Research Center, INF 580, Heidelberg, Germany; 3 Department of Neurology, Hannover Medical School, Hannover, Germany; 4 Proteome Factory AG, Berlin, Germany; University of Florida, United States of America

## Abstract

**Background:**

Amyotrophic lateral sclerosis (ALS) is a fatal disorder of the motor neuron system with poor prognosis and marginal therapeutic options. Current clinical diagnostic criteria are based on electrophysiological examination and exclusion of other ALS-mimicking conditions. Neuroprotective treatments are, however, most promising in early disease stages. Identification of disease-specific CSF biomarkers and associated biochemical pathways is therefore most relevant to monitor disease progression, response to neuroprotective agents and to enable early inclusion of patients into clinical trials.

**Methods and Findings:**

CSF from 35 patients with ALS diagnosed according to the revised El Escorial criteria and 23 age-matched controls was processed using paramagnetic bead chromatography for protein isolation and subsequently analyzed by MALDI-TOF mass spectrometry. CSF protein profiles were integrated into a Random Forest model constructed from 153 mass peaks. After reducing this peak set to the top 25%, a classifier was built which enabled prediction of ALS with high accuracy, sensitivity and specificity. Further analysis of the identified peptides resulted in a panel of five highly sensitive ALS biomarkers. Upregulation of secreted phosphoprotein 1 in ALS-CSF samples was confirmed by univariate analysis of ELISA and mass spectrometry data. Further quantitative validation of the five biomarkers was achieved in an 80-plex Multiple Reaction Monitoring mass spectrometry assay.

**Conclusions:**

ALS classification based on the CSF biomarker panel proposed in this study could become a valuable predictive tool for early clinical risk stratification. Of the numerous CSF proteins identified, many have putative roles in ALS-related metabolic processes, particularly in chromogranin-mediated secretion signaling pathways. While a stand-alone clinical application of this classifier will only be possible after further validation and a multicenter trial, it could be readily used to complement current ALS diagnostics and might also provide new insights into the pathomechanisms of this disease in the future.

## Introduction

Amyotrophic Lateral Sclerosis (ALS) is the most common fatal adult onset motor neuron disorder. Although some interacting pathomechanisms are already known, therapeutic options remain marginal [1], [2].

With ALS diagnosis being based mainly on clinical and electrophysiological examination, serum and CSF analyses are only performed to exclude ALS-mimicking conditions. A major hindrance to the development of novel therapeutic strategies is that diagnosis usually occurs at a late disease stage, limiting the efficacy of neuroprotective approaches. It was shown that up to 10% of ALS patients die before achieving diagnostic certainty according to the revised El Escorial criteria [3], [4].

These factors spur on an intensive search for diagnostic markers to enable earlier ALS detection, and help monitor disease progression and treatment response while furthering the understanding of the associated pathomechanisms. Despite notable contributions of CSF metabolomics profiling in ALS and considerable advances in mapping the normal human CSF proteome, the discovery of ALS-specific protein markers in CSF would allow the most direct clinical application [5]–[7]. In one of the first clinical proteomics studies in this field, Ranganathan et al. used surface-enhanced laser desorption/ionization time-of-flight mass spectrometry (SELDI-TOF-MS) to compare 23 ALS patients with 31 controls and identified three predictive ALS marker proteins [8]. Pasinetti et al. also used SELDI-TOF-MS to study ALS (n = 36) and control (n = 21) CSF. They proposed a combination of proteins as a putative ALS profile [9]. A more recent SELDI technology-based study by Ryberg et al. (2010) profiled CSF of 100 ALS patients and a total of 141 control subjects to build a predictor model based on 41 mostly unidentified masses [10]. However, no independent validation cohort was used to validate these findings.

In our study, primary CSF low molecular weight proteome profiles of 35 ALS patients and 23 age-matched controls were generated. To minimize pre-analytical variability, an immunodepletion of high-abundance CSF proteins was not performed. CSF was processed using chromatographically functionalized paramagnetic beads in combination with linear MALDI-TOF-MS (matrix-assisted laser desorption/ionization) to increase reproducibility, resolution and identification capabilities compared to SELDI-TOF approaches [11]. Multivariate bioinformatic analysis revealed differential masses between ALS and control CSF. Peptide and protein identification by tandem MS enabled us to build a classifier to predict ALS with high sensitivity and specificity. The resulting list of 186 unique CSF proteins was further analyzed using enrichment analysis. Among the 38 classifier masses were ALS biomarker candidates like cystatin C, alpha-1-antitrypsin, VGF, chromogranin A and SPP1 [12]. To evaluate our results in a broader biochemical context, we performed a pathway-based analysis of the identified classifier peptides and proteins. Univariate analysis by mass spectrometry and ELISA confirmed the significant upregulation of secreted phosphoprotein 1 (SPP1) in ALS patients in an independent validation cohort. Moreover, a quantitative Multiple Reaction Monitoring (MRM) mass spectrometry assay was performed for further validation of all markers.

## Materials and Methods

### Patients

**Figure 1:**
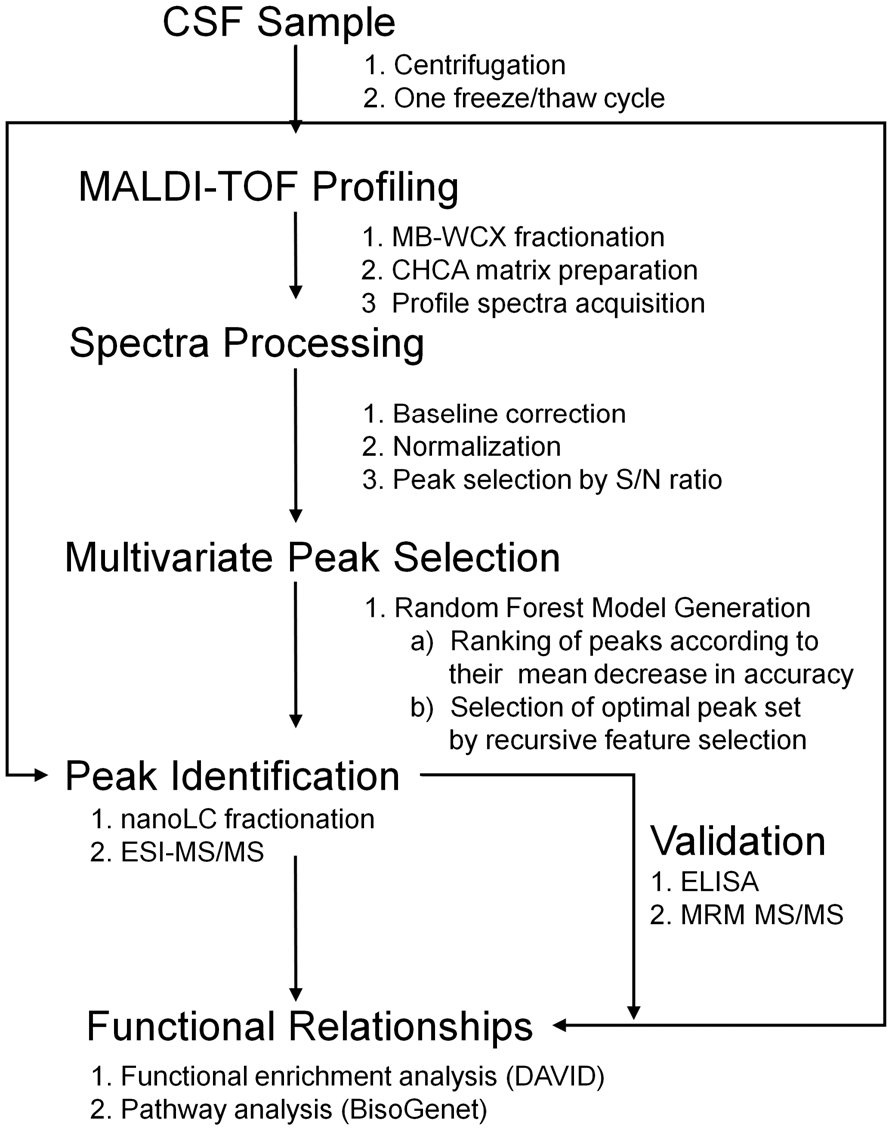
Workflow of CSF profiling by MALDI-TOF MS spectra acquisition and data processing. After analysis by MALDI-TOF mass spectrometry, profile spectra were pre-processed. Discriminatory features were selected using a Random Forest Classifier. Prediction of diagnostic accuracy was assessed by cross-validation. Mass identification of discriminatory mass peaks was enabled by nanoLC-ESI-MS/MS. Validation was performed by ELISA. Further validation was performed using Multiple Reaction Monitoring (MRM) for the quantification of protein expression.

Primary CSF specimens from 35 ALS patients and 23 controls (main patient cohort) were included in this study. The ALS patients were diagnosed according to the revised El Escorial criteria [3]. The group of control subjects consisted of age-matched patients with different neurological diseases, e.g. headache, vertigo, facial nerve paresis, back pain, peripheral neuropathy. No abnormalities were detectable in routine CSF analysis. Relevant patient demographics are summarized in Table 1a. An independent cohort (Table 1b), consisting of primary CSF specimens from 23 ALS patients and 23 controls, was used for validation purposes. After lumbar puncture, all CSF samples were centrifuged to remove cellular debris, frozen on dry ice immediately upon withdrawal in 0.5 mL aliquots and stored at –80°C until further analysis (Figure 1).

**Table 1:** Patient Demographics.

a) Main Cohort		ALS	Control
Samples		35	23
Gender	male	18	11
	female	17	12
Age at CSF collection (years)		59±12.77	62.39±12.24
Disease Duration (months)		13.43±19.72	NA
Onset site (ALS)	limb	23	NA
	bulbar	12	NA
Familial ALS	yes	4	NA
	no	31	NA
Level of diagnostic certainty according to El Escorialcriteria: Possible ALS: Laboratory-supportedprobable ALS: Probable ALS: Definite ALS:		7 (20%) 10 (29%) 13 (37%)5 (14%)	NA
SOD1	yes	0	NA
	no	35	NA
**b) Validation Cohort**		**ALS**	**Control**
Samples		23	23
Gender	male	16	14
	female	7	9
Age at CSF collection (years)		55.91±10.26	55±11.09
Disease Duration (months)		12.57±9.29	NA
Onset site (ALS)	limb	21	NA
	bulbar	2	NA
Familial ALS	yes	1	NA
	no	22	NA
Level of diagnostic certainty according to El Escorialcriteria: Possible ALS: Laboratory-supportedprobable ALS: Probable ALS: Definite ALS:		5 (22%) 6 (26%) 9 (39%)3 (13%)	NA
SOD1	yes	0	NA
	no	23	NA

NA  =  not applicable.

### CSF Preparation with ClinProt Beads and Proteomic Profiling

In contrast to the proprietary chip-based SELDI-TOF MS analysis, we used superparamagnetic particles (ClinProt beads, Bruker Daltonics, Bremen, Germany) to prepare CSF for MALDI-TOF-based proteomic profiling. CSF peptides and proteins were concentrated on the surface of weak cation exchange microparticles (MB-WCX, particle size <1 µm, mean pore size 40 nm; specific surface area 100 cm^2^/g) according to adapted manufacturer’s instructions. The protocol was adapted to 10 µl CSF and binding solution volumes, followed by the addition of 10 µl bead suspension. The dried bead eluates were mixed with alpha-cyano-4-hydroxycinnamic acid matrix solution and spotted onto a MALDI Anchor Chip target (Bruker). The analysis was performed with a linear Microflex MALDI-TOF mass spectrometer (Bruker) optimized for highly sensitive detection in the mass range up to 10 kilodaltons. The raw data used for statistical analysis and detailed patient information are available from http://www.mh-hannover.de/proteomix/plos/. Username and password will be provided upon request.

### Identification of CSF Components

Due to the limited availability of sample material, CSF proteins/peptides from 6 ALS samples and 5 controls were used for peptide identification. The CSF samples were enriched and analyzed in 11 separate nanoLC-ESI-MS/MS high resolution experiments (Proteome Factory AG, Berlin, Germany). Peptide fragmentation and detection were accomplished in the instrument’s LTQ ion trap. Proteins were identified using MS/MS Ion Search of the Mascot search engine (Matrix Science, London, UK) and the Entrez protein database (National Center for Biotechnology Information, Bethesda, MD, USA).

### Functional Enrichment of CSF Components

The identified proteins were further analyzed using enrichment analysis based on the DAVID bioinformatics online resource [13]. Functional groups from Tissue Expression (UP_tissue), Swiss Prot keywords (SP Pir keywords) and Gene Ontology: Molecular Function (GO MF) were assessed for enrichment in CSF.

### Statistical Analysis of MALDI-MS Data

The MALDI-TOF mass profiles were integrated into a multistep bioinformatics analysis. First, spectra were pre-processed using the PROcess R-package [14]. After resampling and baseline correction, normalization was performed using the total ion count. Following quality control, mass spectra from 35 ALS patients and 23 controls (main patient cohort) were included in the final analysis. Peak finding was applied to the mean spectrum of the pre-processed spectra. For classification and peak ranking, we used a Random Forest (RF) predictor [15], [16]. The RF method is an ensemble classifier that consists of a collection of decision trees. Each tree is constructed using a bootstrap subsample of the data. Class assignment for a sample is performed separately for each tree of the collective. The percentage of trees voting for the class of interest is used to define a degree of class membership between 0% and 100% The final class assigned to a sample is determined by major vote (>50%). At each iteration (bootstrap subsampling) of the RF construction, the data that were not part of the training subsample (out of bag data) make it possible to estimate the error rate. The average (mean) error over all iterations is commonly referred to as the out of bag error (OOB). Peak importance can be estimated using the mean decrease in accuracy (MDA) measure. This gives the increase in OOB error when the OOB data for that peak are permuted while all others are left unchanged. For an unbiased estimation of classifier performance on new samples, we used 10-fold cross-validation, selecting each sample 10 times for the test set. All feature ranking/selection steps using class information were included in the cross-validation. Generalization performance was visualized using a ROC curve [17]. To find an optimal peak set, we used recursive feature elimination, at each step removing the peaks with a mean decrease in accuracy lower than the median mean decrease in accuracy [18]. The peak sets were rated according to their cross-validation area under the curve (AUC), choosing the set with the highest AUC for further analysis. The AUC was calculated using the percentages of ensemble trees voting for a specific class. Accuracy, sensitivity and specifity were calculated based on class assignment according to major vote. Statistical significance of differential expression between different groups was calculated using the Wilcoxon rank test (two-tailed). All statistical tests were carried out using the open source software R (http://www.r-project.org). P-values of p< = 0.05 were considered significant, while p-values of p< = 0.1 were considered to show a trend towards statistical significance. The reported fold changes for the respective proteins of interest were calculated by dividing their mean concentrations in the ALS samples by the mean concentrations in the controls.

### ELISA Validation

The expression of SPP1 was validated in the validation cohort using ELISA technology according to the manufacturer’s instructions (RayBiotech Inc., Norcross, GA, USA) at a 75-fold dilution. CSF samples were randomly assayed in duplicate. Inter-assay variation was minimized by measuring three samples on different ELISA plates and subsequent normalization.

### Multiple Reaction Monitoring (MRM) for the Quantification of Protein Expression

Eight ALS, four of our controls and four additional control CSF samples (NextGen sample ID no. 101, 102, 103, 104), were tested against the CSFassay-human A.1.0 protein assay (NextGen Sciences, Inc, Ann Arbor, MI, USA) [19]. Briefly, CSF samples (50 µl) were processed to generate tryptic peptides using a proprietary sample processing method developed by NextGen Sciences. The resulting peptide-containing solutions were spiked with known quantities of heavy stable-isotope labeled (heavy) peptides that have identical sequences and are chemically equivalent to the endogenous (light) peptides. The spiked samples, calibrators and quality controls were injected to acquire data by LC-MRM/MS using a 25 minute scheduled method. MRM data were processed to obtain absolute quantitation results using NextGen Sciences’ proprietary software. Protein quantity was expressed in [ng/ml] and subsequently transferred to statistical analysis. Down or upregulation of protein concentrations was described as a fold change (FC) value. FC values <1 mean downregulated and >1 upregulated in the ALS CSF samples.

## Results

Cerebrospinal fluid contains a high diversity of proteins, 186 of which were identified in this study (Table S1). While 44 of the CSF proteins could be identified in both the 6 analyzed ALS and the 5 control samples, 62 were only identified in ALS and 80 in control CSF (Figure S1). Comprehensive Mascot search results are available from http://www.mh-hannover.de/proteomix/plos/mascot. Username and password will be provided upon request. Performing a DAVID analysis on the full set of identified proteins, several functional groups were found to be significantly enriched in CSF (Table 2a and 2b).

**Table 2:** DAVID analysis of the full set of identified proteins in CSF.

a) Significantly Enriched UP Tissue Expression		
UP Tissue	%	P-value (BH corrected)
Brain	63.21	<0.001
Plasma	10.38	<0.001
Bile	4.72	<0.001
Epithelium	27.36	<0.001
Saliva	4.72	<0.001
Liver	22.64	<0.001
Cajal-Retzius cell	6.60	0.001
Fetal liver	6.60	0.001
Platelet	9.43	0.003
Mesangial cell	2.83	0.004
		
**b) Significantly Enriched Gene Ontology: Molecular Function**		
**GO Molecular Function**	**%**	**P-value (BH corrected)**
GO:0008092∼cytoskeletal protein binding	13.208	0.006
GO:0003779∼actin binding	10.377	0.007
GO:0060228∼phosphatidylcholine-sterol O-acyltransferase activator activity	2.830	0.034
GO:0030414∼peptidase inhibitor activity	6.604	0.032

**Figure 2:**
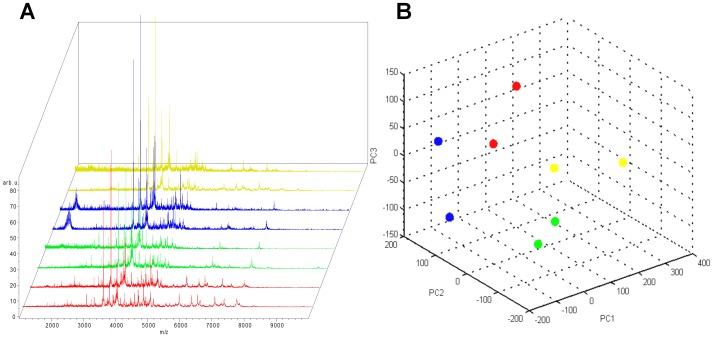
Reproducibility of MALDI-TOF profile spectra. Principal component analysis (PCA) for the entire spectra (1–10 kDa) of four different samples using the ClinProTools Software (Version 2.1, Bruker Daltonics). A: Replicate spectra from one sample are colored red, green, blue and yellow in the stack view. B: Each dot in the PCA plot represents one technical replicate from two repeated sample preparations over the course of one month. Despite inevitable variations in ambient and instrument conditions, the replicates form distinct clusters according to their biological origin.

To screen for differentially regulated proteins and peptides that may serve as biomarkers for ALS, we used weak cationic exchange magnetic beads to enrich a protein and peptide fraction of interest [20]. This allows for the depletion of some abundant CSF proteins, a common approach in proteomic screening, while minimizing the inadvertent removal of less abundant potential biomarkers in pre-fractionation procedures [21]. The eluted fractions were analyzed with a linear MALDI-TOF-MS, facilitating the acquisition of a set of profile spectra in the mass range below 10 kDa (Figure 2). Using this approach, the peak recognition algorithm was able to detect 153 mass peaks. Using the detected peaks, we were able to classify the spectra according to their ALS status. Using all detected peaks in the classifier construction process, the following prediction performance was achieved: accuracy: 77.2% (448/580); sensitivity: 78% (273/350); specificity: 76.1% (175/230); area under the curve (AUC): 0.82. Using recursive feature elimination to search for the optimal peak set, the biomarker set was reduced to the top 25% of 153 peaks (Table 3).

**Table 3:** Top Peaks Used for Classification.

Peak Mass [m/z]	Mean Decrease inAccuracy (z-Score)	Protein ID	Peptide Peaks Matched	Protein Identity GI #	Protein Mass [Dalton]	MASCOT Score
8398.49	7.66497					
9346.98	5.61487					
2116.79	4.19806					
2378.33	2.86895	alpha-1 antitrypsin	2	177816	3724	58
9738.48	2.79567					
9061.22	1.52224					
8633.19	1.30805					
7453.3	1.24643					
9635.76	1.0146					
4008.93	0.87181					
1213.53	0.82527					
4353.34	0.72187					
4283.89	0.69717					
9134.49	0.65734					
1315.84	0.56229	cystatin-C precursor	4	4503107	15789	128
2069.8	0.51385					
3954.58	0.48911	VGF nerve growth factorprecursor	13	17136078	67218	220
4092.77	0.43934					
3807.59	0.43499					
8798.94	0.4059					
3253.32	0.40418					
3405.3	0.37003	VGF nerve growth factorprecursor	13	17136078	67218	220
3023.91	0.34859					
3689.73	0.34071	VGF nerve growth factorprecursor	13	17136078	67218	220
3380	0.33391					
1270.6	0.33247					
1401.38	0.31381					
3909.19	0.304	chromogranin A	2	2072129	50688	61
5067.31	0.27799					
1298.07	0.26727					
6690.88	0.18376					
3514.26	0.18011					
6125.72	0.14527					
1676.56	0.13198					
1541.13	0.13908	secreted phospho-protein 1 isoform b	2	4759166	33823	88
9211.54	0.11068					
7743.15	0.10234					
1520.48	0.08217					

**Figure 3:**
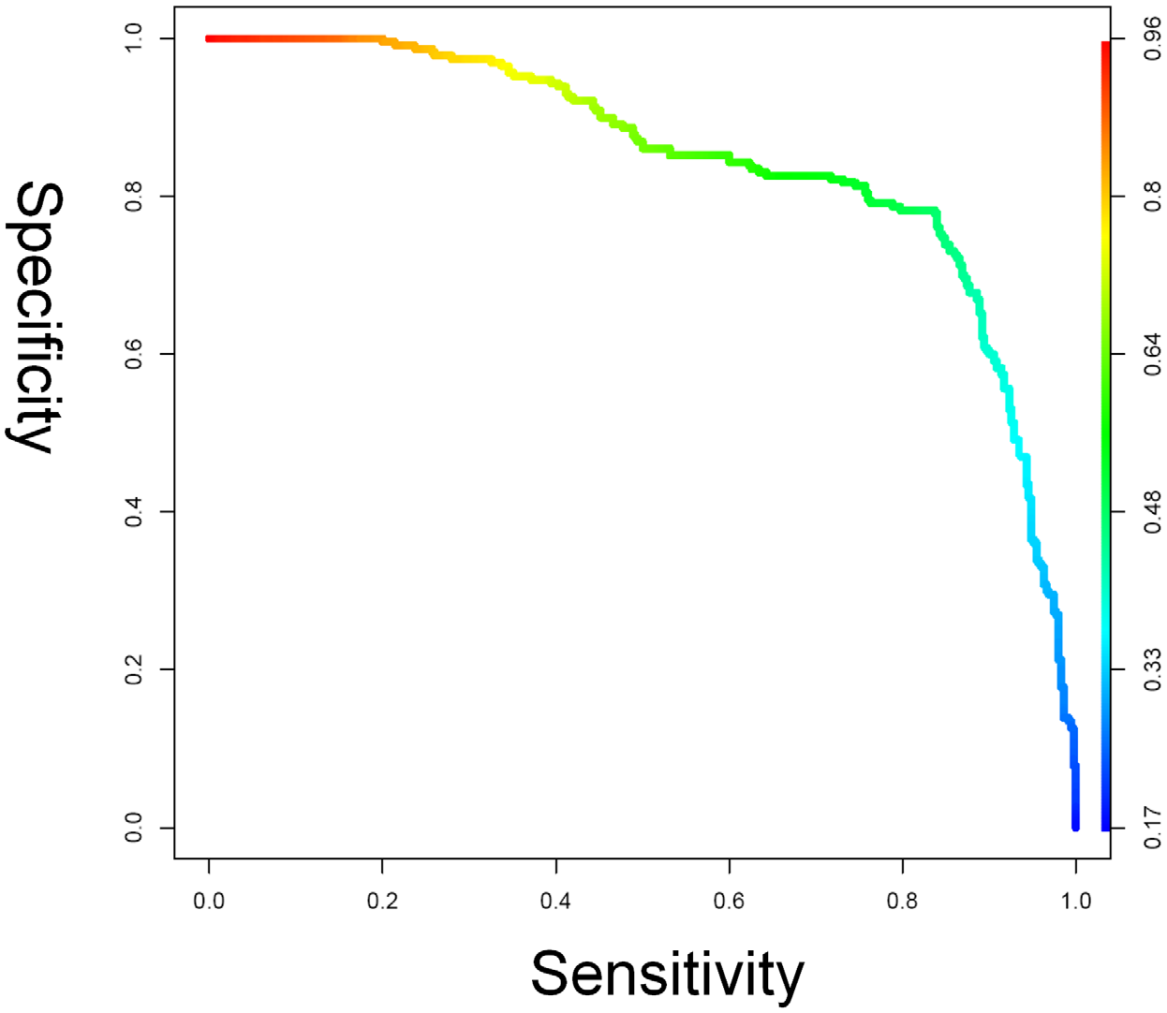
ROC plot analysis with area under the curve of CSF classification of ALS/non-ALS spectra. The Random Forest and decision tree predictor assign a score between 0 ( = non-ALS) and 1 ( = ALS) to all spectra. A CSF sample that shows a higher score than a predefined cut-off is classified as an ALS sample. The ROC plot curve shows the sensitivity against the specificity for different cut-offs. The curves are color-coded according to the level of the cut-off. The results shown are for the Random Forest with the optimized set of 38 peaks.

These top peaks not only manage to retain the data partition achieved using all peaks, but also show a better generalization performance. The prediction performance was: accuracy: 80.3% (466/580); sensitivity: 80.7% (286/350); specificity: 78.3% (197/250); AUC: 0.84. The results for the feature optimized model are presented as a receiver operating characteristic curve (ROC) in Figure 3. Using ESI-MS/MS, we identified some of the top classifying peaks (Table 3) in selected samples.

The proteins in the CSF matching the MALDI-TOF-MS mass peaks were alpha-1-antitrypsin (A1AT), cystatin C (CST 3), chromogranin A (CHGA), VGF nerve growth factor inducible protein (VGF) and the secreted phosphoprotein 1 (SPP1).

**Figure 4:**
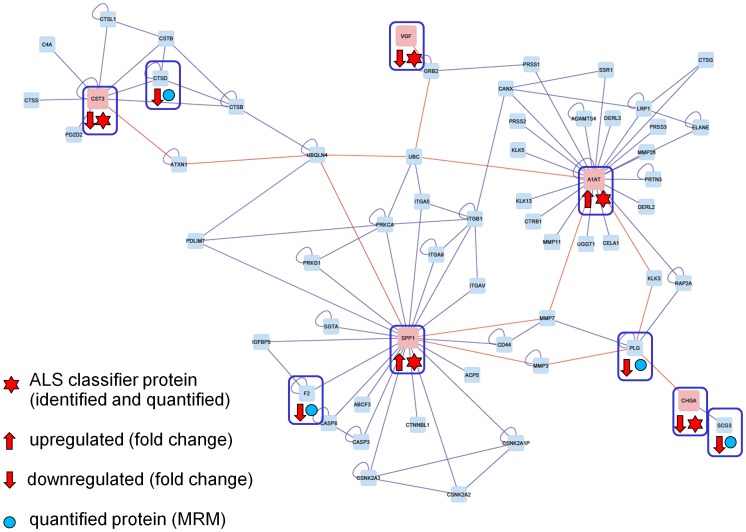
BisoGenet Pathway. The pathway shown was generated using BisoGenet [22]. It shows the biomolecular relationships involving A1AT (alpha-1 antitrypsin; FC: 0.96), CST3 (cystatin-C precursor; FC: 0.96), CHGA (chromogranin A; FC: 0.90), VGF (nerve growth factor precursor; FC: 0.79) and SPP1 (secreted phospho-protein 1 isoform b; FC: 0.94). The red lines mark the shortest paths between the identified biomarker candidates which are represented by red nodes. FC: Fold Change.

**Figure 5:**
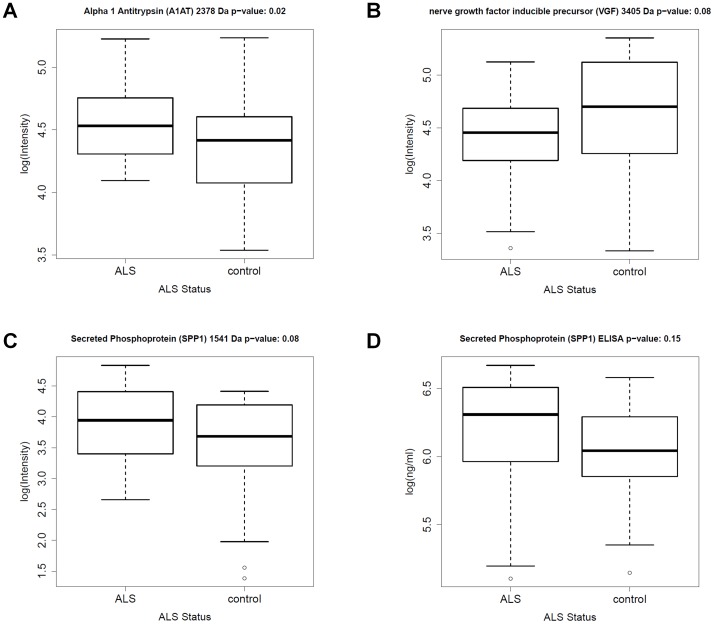
Boxplots. The four boxplots visualize the expression of A: A1AT, B: VGF 3405 Da peptide, C: SPP1 in the main patient cohort and D: SPP1 in the validation cohort as quantified by ELISA.

To evaluate our results in a broader biochemical context, we performed a pathway-based analysis of the identified classifier peptides and proteins. The BisoGenet multi-tier Cytoscape application was used for visualization and analysis of biomolecular relationships involving A1AT1, CST3, CHGA, VGF and SPP1 [22]. The resulting protein network is shown in Figure 4. Our biomarker panel allowed the detection of ALS using a multivariate classification model. Mass peaks from the optimized peak set and corresponding peak intensities in ALS patients and control samples (see Figure 5 and Figure S2 for boxplots) were further evaluated individually, using the Wilcoxon rank test (two-tailed).

A1AT (p-value = 0.02) was found to be significantly (p< = 0.05) upregulated in ALS patients compared to the controls. Further, there was a trend (p< = 0.1) towards a lower expression of VGF, cystatin C (CST 3) and chromogranin A (CHGA). A higher expression of secreted phosphoprotein 1 (SPP1, p-value = 0.08) was detectable in the ALS patients. VGF was already identified as potential biomarker in ALS, [9] while an upregulation of SPP1 has previously been reported in the microglia of transgenic ALS mice [23]. Thus, to assess the differential expression of SPP1 in ALS patients, an ELISA was performed using 46 the CSF samples from our validation patient cohort (Table 1b). The SPP1 ELISA confirmed an upregulation of SPP1 in ALS patients (Figure 5).

Since a CSF ELISA assay was only available for SPP1, we decided to further validate our findings in a quantitative mass spectrometry assay. (CSF assay-human A.1.0 protein assay, NextGen Sciences). This assay allows the simultaneous measurement of the concentration of 80 proteins in human CSF samples. We used this procedure for 16 CSF samples. From the validation patient cohort, 8 ALS patient CSF samples were run together with four of our control samples and four normal human CSF controls (NextGen) in a single batch. All controls met precision specification goals of <30%. The results for our marker panel consisting of A1AT, CST 3, CHGA, and VGF were in line with the MALDI-TOF-MS data-derived ALS classifier. The fold changes in concentration for these proteins as well as for the 75 remaining analytes are listed in Table S2 which also shows how the MRM results are complemented by the MS/MS peptide identifications and their alignment with the BisoGenet pathway. Apart from our marker proteins, the MRM assay revealed 4 additional differentially expressed proteins which are a part of the biomolecular network (Figure 4). These proteins were cathepsin D (CTSD), thrombin (F2), plasminogen (PLG) and secretogranin III (SCG3). All 4 proteins were slightly downregulated in the ALS patients.

## Discussion

A definite diagnosis of ALS can, according to the diagnostic criteria in current use, be reliably made only in advanced disease stages. Laboratory and technical tests can help to exclude other ALS-mimicking syndromes but do not provide positive markers of the disease. It is therefore necessary to improve the sensitivity and specificity of the diagnostic tools. Moreover, the identification of disease-specific markers and their interaction in a common systems biological network could allow novel insights into disease-associated dysregulation of signalling cascades and more accurate monitoring of patients within therapeutic trials.

Proteomics represents a combination of different methods for the study of peptides and proteins in tissues, biological fluids or cell cultures at a specific point in time [22], [24], [25]. In context with diseases such as ALS, protein expression analysis means the detection of specific protein changes in affected cells or in body fluids such as CSF [8], [22], [24].

ALS is a degenerative disorder of the central nervous system which primarily affects motor neurons in brain and spinal cord even though more widespread changes in brain regions other than the primary motor cortex have been detected by neuropathological and neuroimaging techniques. CSF may most probably reflect biochemical disease-related alterations as it is much closer to the central motor system than blood or urine. Analysis of the CSF proteome of ALS patients can identify pathophysiologically relevant pathways as well as non-specific changes associated with neurodegeneration in general. The protein component of CSF not only consists of brain-derived proteins but also of proteins abundant in plasma [26]. Differences found in CSF may therefore result from increased or reduced expression/turnover in neurons and glial cells, sequestration into intracellular protein aggregates, but also from dysfunction of the blood-brain-barrier and subsequent entry of proteins from the plasma [27]–[29]. For the identification of differentially expressed peptides and proteins, chip or paramagnetic bead-based methods in combination with MALDI-TOF-MS can be performed. Using this approach, we and others [8]–[10] were able to find differences in the CSF proteome of patients with ALS and to identify disease-specific biomarkers or changes in protein patterns. CSF contains a large number of different peptides and proteins with a highly variable composition. Therefore, advanced pattern recognition algorithms are used in this study to associate these peptide and protein fingerprints with known disease states, resulting in the identification of unique protein patterns that can distinguish between healthy and affected individuals. We used a robust WCX bead-based MALDI-TOF platform in combination with the Random Forest algorithm [15] to deal with the complexity of the low molecular weight CSF proteome. This enabled us to select peaks not only based on their own expression changes, but also evaluating them directly in the context of complex interactions and composition changes. This made it possible to extract the most important information from the complex patterns for the classification model. The complexity of the spectra also makes cross-validation necessary to get an unbiased estimate of classifier performance on new samples. In our study, we identified five classifier proteins that played a predictive role in our optimized ALS biomarker network: A1AT, CST3, CHGA, VGF and SPP1. These findings for A1AT, CST3, CHGA and VGF are in line with previously published data [9], [24].

Cystatin C is widely expressed in neuronal and non-neuronal cells and physiologically acts as an inhibitor of extracellular cystein proteases. Its concentration in the CSF is about 5-fold higher than in serum, mainly resulting from secretion by the choroid plexus [30], [31]. Cystatin C prevents proteolytic cleavage resulting from leakage of cysteine proteases from damaged cells but its exact role in ALS or other neurological conditions still remains unclear as both neurotoxic and neuroprotective effects have been described [32], [33]. It is - together with transferrin - one of the two protein components of Bunina bodies, small eosinophilic intracytoplasmic inclusions considered as a neuropathological hallmark of ALS [34]–[37]. A recent study has specifically investigated whether cystatin C content in ALS CSF (measured by ELISA) could serve as a disease biomarker: the authors describe a correlation between lower Cystatin C levels and shorter survival times as well as significantly reduced levels in ALS patients in comparison to healthy controls but not to CSF from patients with other neurological disorders, again highlighting the benefit of biomarker panels consisting of several differentially regulated peptides [12].

VGF and CHGA belong to the chromogranin/secretogranin family of regulated neurosecretory proteins which can be detected in secretory large dense-core vesicles (LDCV) in neurons and neuroendocrine cells [38]. Granins are pro-hormones and their cleavage products possess Ca2+ binding, protein sorting and antibiotic properties and are involved in the regulation of the cellular content of Ca2+, hormones, ATP and catecholamines [39]. VGF has been identified as a potential ALS biomarker using SELDI-TOF-MS [9]. In a follow-up study, VGF levels in CSF were shown to correlate with progressing muscle weakness [40]. Decreased VGF content in the CSF has also been described in Alzheimer’ disease and frontotemporal dementia [41], [42] . VGF expression increases with neuronal activity, decreases with aging and is regulated by neurotrophic factors such as brain-derived neurotrophic factor (BDNF) [43] . The decrease in VGF could therefore be the result of either neuronal loss/dysfunction and/or reduced growth factor secretion. In a transgenic ALS mouse model, VGF expression was reduced already in presymptomatic stages and decreased further with disease progression. *In vitro,* adenoviral VGF overexpression was protective against excitotoxic injury of primary spinal cord neurons [40]. A role of chromogranins in the pathophysiology of both familial and sporadic ALS is supported by studies in ALS transgenic mice: CHG A and B selectively co-localize and interact with misfolded mutant SOD1 in spinal cord motor neurons and interneurons. This triggers the secretion of mutant SOD1 into the extracellular space where it induces microgliosis and subsequent motor neuron death [38]. Given the fact that we studied CSF of patients with sporadic ALS (sALS), it is of particular interest that wild-type SOD1, once oxidized by hydrogen peroxide, also interacts with CHGB, can be secreted and induces microglial activation and dose-dependent death of motor neurons [44]. This corresponds with a recent immunohistochemical study in spinal cord tissue of sporadic ALS (SALS) patients, where CHGA and CHGB displayed a characteristic pattern in motor neurons with intracellular aggregation and colocalization of chromogranins together with SOD1 [45]. Alterations in CHGA and B expression and localization, however, do not seem to be ALS-specific. They have also been detected in plaque in brain tissue of patients with Alzheimer’s disease and Creutzfeldt-Jacob disease [46]–[48]. Reduced CHGA (as well as VGF and cystatin C) levels were found in a proteomic analysis of CSF of patients with Alzheimer’s disease [49].

Alpha-1-antitrypsin is part of the serine protease inhibitor (serpin) family. It has been detected in amyloid plaques of Alzheimer’s disease patients, most probably as a result of increased astrocytic secretion [50]. CSF upregulation of alpha-1 antitrypsin was previously found in ALS by two-dimensional gel electrophoresis-based studies, similar to our observation of increased A1AT content in ALS-CSF [27]. It was also shown to be increased in CSF and plasma in various other neurological diseases ranging from Alzheimer’s disease to Guillain-Barre syndrome and is now mainly interpreted as an epiphenomenon due to blood brain barrier (BBB) dysfunction [51]–[53].

SPP1 is an extracellular matrix protein first identified in osteoblasts but also expressed in neurons and glial cells. It is upregulated in acute and chronic inflammation, has been shown to possess neuroprotective capacities and was identified as a biomarker in the plasma of patients with relapsing-remitting multiple sclerosis [54], [55]. In mutant SOD1-G93A transgenic ALS mice, increased SPP1 expression was measured in spinal cord microglia, possibly contributing to the beneficial effects of neuroinflammation [23].

Our study is therefore in line with previous findings in the CSF of not only ALS but also other degenerative diseases of the CNS. Therefore, it remains to be shown whether these members of our biomarker panel are specific for ALS or rather unspecific for different diseases associated with neuronal degeneration. As assessed by univariate analysis, two of the identified biomarker proteins (A1AT and SPP1) individually showed a significant change of expression in ALS patients.

Although suspected to be non-specifically affected by different neurological diseases - diminishing their potential value as singular biomarkers for ALS - A1AT, CHGA, VGF and cystatin C together do, however, appear to be highly specific for ALS detection as a part of the condensed marker panel described in this study.

Using a new mass spectrometry-based technique (MRM) for the quantitation of peptides and proteins in the CSF of our ALS patients, we were able to identify other proteins like cathepsin D (CTSD), thrombin (F2), plasminogen (PLG) and secretogranin III (SCG3) as key players in the biomarker network (Figure 4). These proteins have no classifying function concerning the biomarker panel of this study. Nevertheless, we could show that they interact with the five proteins of our biomarker panel.

CTSD is also a member of the cystatin family [56]. In our network, we could show an interaction with the upregulated classifier protein SPP1. Thrombin acts via protease-activated receptors on the platelet cell membrane, promoting platelet activation and aggregation. Platelets have been shown to be systemic markers for disease in several neurological disorders, including ALS [57].

A further downregulated protein was SCG3 [58]. Like chromogranin A, it also belongs to the granin/secretogranin family [30] and like the classifier protein chromogranin A, it is also downregulated concerning the MRM analysis. We also identified plasminogen (PLG) in our study. This protein interacts directly with the other downregulated proteins CHGA and SCG. PLG belongs to the serin protease family and can be activated by the urokinase-type plasminogen activator (uPA) system. In 2007, Glas et al showed that the inhibition of the uPA system prolonged the life of transgenic animals and that the overexpression of PLG may therefore play a role in the complex pathogenesis of ALS [59].

Further experiments with a strong aspect of quantitative analysis will be necessary to obtain a better understanding of the complex interactions involved in the onset of ALS. Moreover, to become useful as a diagnostic tool, CSF samples of patients in earlier disease stages and of patients suffering from ALS-mimicking conditions will have to be analyzed.

In summary, this study shows that CSF proteome profiling using bead-based protein fractionation and MALDI-TOF mass spectrometry in combination with specialized data mining tools is able to identify biomarker patterns which have similarly been described in previous studies analyzing ALS CSF. While further studies are required to better understand the disease specificity of these markers, they ultimately might become valuable for improved diagnosis of ALS, monitoring of patients in clinical trials and increased understanding of underlying pathomechanisms. We built a validated protein network that will enable us to gain a deeper understanding of the complex nature of this disease. To identify enriched functionally-related protein groups, cluster these groups in terms of subcellular localization and map enriched protein groups to KEGG pathways, a DAVID analysis was performed for the 59 peptides and proteins that make up our pathway analysis-derived ALS proteome network. The results of this analysis are shown in Table S3. Interestingly, the most enriched protein groups were mapped to the neuroactive ligand-receptor interaction, lysosome and focal adhesion KEGG pathways, which further underlines the potential significance of our results in the quest to discover ALS-specific disease mechanisms [13]. The flexibility of our approach will also provide the possibility to integrate new findings from different databases and new proteomic data into our biomarker network (Figure 4). Our results emphasize that it is essential to employ a biomarker panel and a biomarker network. In contrast to the poor specificity of individual cross-disease biomarkers, an interaction map of this condensed panel may provide a better insight into deregulated biochemical pathways involved in ALS.

## Supporting Information

Figure S1 **Venn diagram of identified CSF proteins.** The Venn diagram shows that 44 of the 186 CSF proteins identified in this study were identified in ALS and control CSF alike while 62 were only identified in ALS and 80 in control samples.
(TIFF)

Figure S2 **Boxplots.** The four boxplots visualize the expression of A: CHGA, B: CST3, C: VGF 3689 Da peptide and D: VGF 3954 Da peptide in the main patient cohort.
(TIFF)

Table S1 **CSF proteins identified by high-resolution LC-ESI-MS/MS.**
(DOC)

Table S2 **Comparison between MRM, MS/MS and BisoGenet pathway.**
(PDF)

Table S3 **DAVID analysis of the 59 proteins from the BisoGenet pathway.**
(DOC)
