# Supplementary figures and images for: Monitoring CSF Proteome Alterations in Amyotrophic Lateral Sclerosis: Obstacles and Perspectives in Translating a Novel Marker Panel to the Clinic
Source: PLoS One. 2012 Sep 6;7(9):e44401. doi: 10.1371/journal.pone.0044401 (PMC3435306; doi:10.1371/journal.pone.0044401)

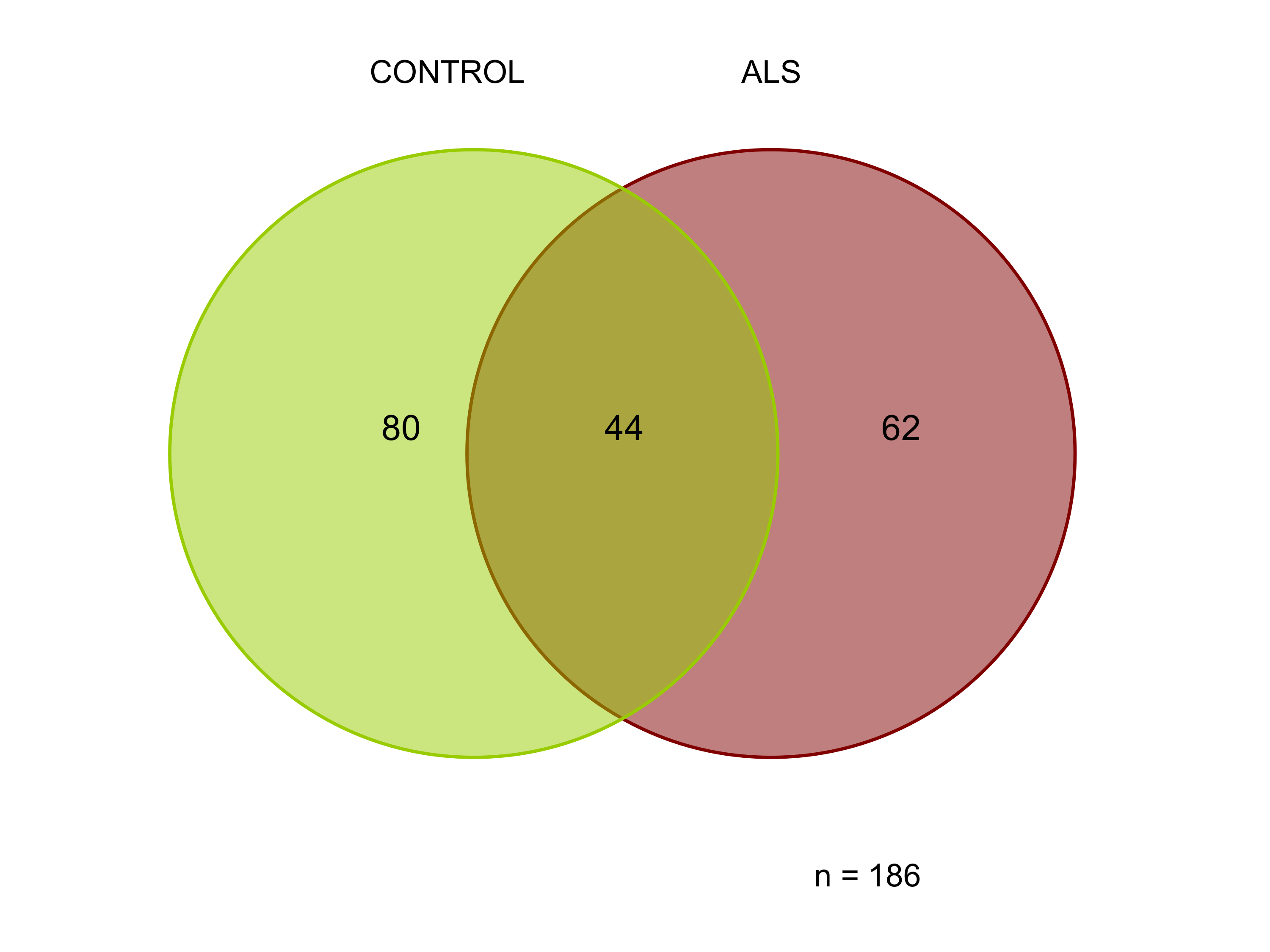

Supplement: Figure S1 — Venn diagram of identified CSF proteins. The Venn diagram shows that 44 of the 186 CSF proteins identified in this study were identified in ALS and control CSF alike while 62 were only identified in ALS and 80 in control samples. (TIFF) [file pone.0044401.s001.tiff]

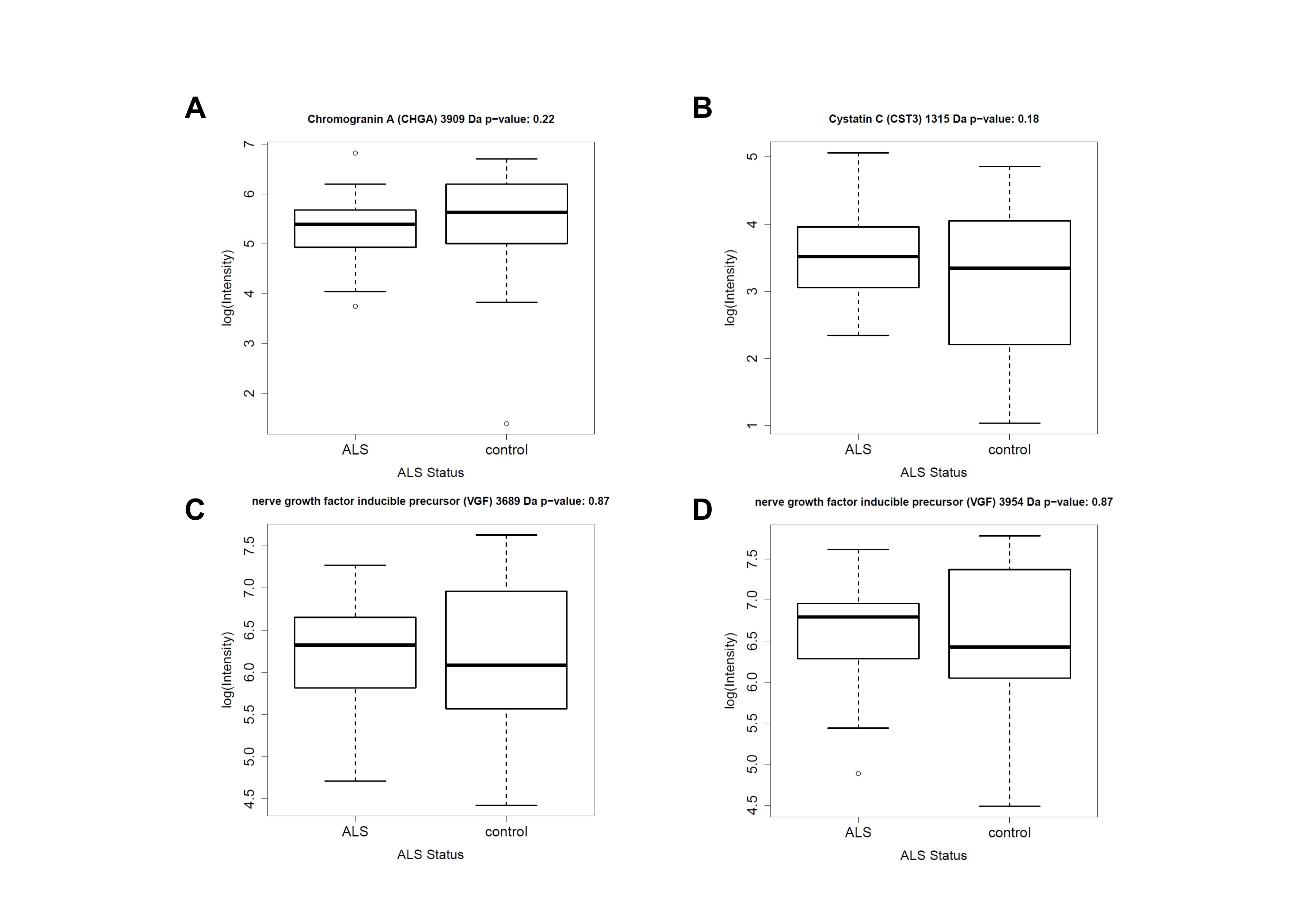

Supplement: Figure S2 — Boxplots. The four boxplots visualize the expression of A: CHGA, B: CST3, C: VGF 3689 Da peptide and D: VGF 3954 Da peptide in the main patient cohort. (TIFF) [file pone.0044401.s002.tiff]
