# Supplementary material for: Monitoring CSF Proteome Alterations in Amyotrophic Lateral Sclerosis: Obstacles and Perspectives in Translating a Novel Marker Panel to the Clinic
Source: PLoS One. 2012 Sep 6;7(9):e44401. doi: 10.1371/journal.pone.0044401 (PMC3435306; doi:10.1371/journal.pone.0044401)
Supplement: Table S1 — CSF proteins identified by high-resolution LC-ESI-MS/MS. (DOC) [file pone.0044401.s003.doc]

**Table S1**

CSF Proteins Identified by High-Resolution LC-ESI-MS/MS

| **#** | **Protein Description** | **Protein Identity GI #** |
| --- | --- | --- |
| 1 | actin nucleation promoting factor [Homo sapiens] | 161376482 |
| 2 | activin type I receptor [Homo sapiens] | 338215 |
| 3 | ALB protein [Homo sapiens] | 23241675 |
| 4 | albumin isoform CRA_a [Homo sapiens] | 119626064 |
| 5 | albumin isoform CRA_b [Homo sapiens] | 119626065 |
| 6 | albumin isoform CRA_h [Homo sapiens] | 119626071 |
| 7 | albumin isoform CRA_n [Homo sapiens] | 119626077 |
| 8 | albumin isoform CRA_t [Homo sapiens] | 119626083 |
| 9 | albumin-like [Homo sapiens] | 763431 |
| 10 | ALL-1 fusion partner from chromosome 6 [Homo sapiens] | 430994 |
| 11 | alloalbumin Venezia [Homo sapiens] | 178345 |
| 12 | alpha-1-antitrypsin (aa 268-394) [Homo sapiens] | 24438 |
| 13 | alpha-1-antitrypsin (alpha-1-AT) [Homo sapiens] | 177816 |
| 14 | amyloid-like protein 1 isoform 2 precursor [Homo sapiens] | 4885065 |
| 15 | antithrombin III [Homo sapiens] | 179161 |
| 16 | apolipoprotein A-II preproprotein [Homo sapiens] | 4502149 |
| 17 | apolipoprotein D apoD [human plasma Peptide 246 aa] | 619383 |
| 18 | apolipoprotein E [Homo sapiens] | 178849 |
| 19 | apolipoprotein J precursor [Homo sapiens] | 178855 |
| 20 | beta-2 microglobulin [Homo sapiens] | 34616 |
| 21 | beta-2-glycoprotein I apolipoprotein H [Homo sapiens] | 28810 |
| 22 | beta-trace protein prostaglandin D synthase PGD synthase {EC 5.3.99.2} [human cerebrospinal fluid peptide 168 aa] | 404390 |
| 23 | brefeldin A resistant Arf-guanine nucleotide exchange factor 1 [Homo sapiens] | 237700723 |
| 24 | calcium channel voltage-dependent P/Q type alpha 1A subunit isoform CRA_a [Homo sapiens] | 119604767 |
| 25 | cat eye syndrome chromosome region candidate 6 isoform b [Homo sapiens] | 251823730 |
| 26 | centromere protein F [Homo sapiens] | 55770834 |
| 27 | CGI-50 protein [Homo sapiens] | 4929569 |
| 28 | chain A apo-human serum transferrin (glycosylated) | 110590599 |
| 29 | chain a apo-human serum transferrin (non-glycosylated) | 110590597 |
| 30 | chain a crystal structure of Cys10 sulfonated transthyretin | 126030594 |
| 31 | chain a crystal structure of human serum albumin | 3212456 |
| 32 | chain a crystal structure of the Ga module complexed with human serum albumin | 55669910 |
| 33 | chain A crystal structure of the R124a mutant of the N-lobe human transferrin | 29726965 |
| 34 | chain A crystal structure of transthyretin mutant I84s at acidic Ph | 126030508 |
| 35 | chain A human serum albumin complexed with myristate and aspirin | 122920512 |
| 36 | chain A human serum albumin complexed with myristate and azapropazone | 78101694 |
| 37 | chain A human serum albumin mutant R218h complexed with thyroxine (3.3'.5.5'-tetraiodo-L-thyronine) | 31615330 |
| 38 | chain A human transferrin N-lobe mutant H249e | 6980467 |
| 39 | chain A structure of prealbumin secondary tertiary and quaternary interactions determined by Fourier refinement at 1.8 angstroms | 230651 |
| 40 | chain A tertiary structures of three amyloidogenic transthyretin variants and implications for amyloid fibril formation | 3891560 |
| 41 | chain A the intact and cleaved human antithrombin Iii complex as a model for serpin-proteinase interactions | 999513 |
| 42 | chain A the X-ray crystal structure refinements of normal human transthyretin and the amyloidogenic Val30met variant to 1.7 angstroms resolution | 443295 |
| 43 | chain I crystal structure of the antithrombin-S195a factor Xa-pentasaccharide complex | 109157826 |
| 44 | chain I P14-fluorescein-N135q-S380c-antithrombin-Iii | 8569387 |
| 45 | chromogranin A | 2072129 |
| 46 | CLL-associated antigen KW-13 [Homo sapiens] | 19851921 |
| 47 | complement cytolysis inhibitor precursor [Homo sapiens] | 180620 |
| 48 | cystatin C [Homo sapiens] | 181387 |
| 49 | EDAR-associated death domain isoform CRA_a [Homo sapiens] | 119590453 |
| 50 | enolase [Homo sapiens] | 31179 |
| 51 | epithelial splicing regulatory protein 2 [Homo sapiens] | 45935393 |
| 52 | espin isoform CRA_a [Homo sapiens] | 119591941 |
| 53 | facioscapulohumeral muscular dystrophy [Homo sapiens] | 1435038 |
| 54 | FBLIM1 protein [Homo sapiens] | 18044300 |
| 55 | F-box only protein 11 isoform 2 [Callithrix jacchus] PREDICTED | 296223905 |
| 56 | F-box protein 11 [Homo sapiens] | 56405842 |
| 57 | fibrinogen alphaA | 223918 |
| 58 | fibrinopeptide A | 229185 |
| 59 | formin binding protein 4 isoform CRA_a [Homo sapiens] | 119588290 |
| 60 | formin-like protein 1 [Homo sapiens] | 33356148 |
| 61 | functional smad suppressing element 18 [Homo sapiens] PREDICTED | 239757043 |
| 62 | gap junction delta-3 protein [Homo sapiens] | 148839378 |
| 63 | hCG1642841 [Homo sapiens] | 119600038 |
| 64 | hCG19253 isoform CRA_a [Homo sapiens] | 119597391 |
| 65 | hCG19253 isoform CRA_c [Homo sapiens] | 119597393 |
| 66 | hCG1990724 isoform CRA_b [Homo sapiens] | 119588659 |
| 67 | hCG1998636 isoform CRA_b [Homo sapiens] | 119572565 |
| 68 | hCG2004654 [Homo sapiens] | 119572417 |
| 69 | hCG2036676 [Homo sapiens] | 119612677 |
| 70 | hCG2040174 [Homo sapiens] | 119589870 |
| 71 | hCG2042958 isoform CRA_e [Homo sapiens] | 119621713 |
| 72 | hCG2045367 [Homo sapiens] | 119600090 |
| 73 | hCG2045936 [Homo sapiens] | 119572890 |
| 74 | hCG22358 isoform CRA_a [Homo sapiens] | 119601600 |
| 75 | hCG38312 isoform CRA_c [Homo sapiens] | 119614331 |
| 76 | homeobox protein DLX-6 [Homo sapiens] | 294610640 |
| 77 | huntingtin [Homo sapiens] | 4586876 |
| 78 | hypothetical protein [Homo sapiens] | 50949458 |
| 79 | hypothetical protein [Homo sapiens] | 51476390 |
| 80 | hypothetical protein [Homo sapiens] | 7328064 |
| 81 | hypothetical protein LOC168850 isoform CRA_c [Homo sapiens] | 119604033 |
| 82 | hypothetical protein LOC65998 [Homo sapiens] | 222144283 |
| 83 | IDN4-GGTR6 [Homo sapiens] | 4760539 |
| 84 | Ig A L | 229536 |
| 85 | Ig lambda C | 223815 |
| 86 | insulin receptor substrate 4 [Homo sapiens] | 4504733 |
| 87 | KIAA0029 [Homo sapiens] | 40788955 |
| 88 | KIAA0346 [Homo sapiens] | 20521009 |
| 89 | KIAA0360 [Homo sapiens] | 20521013 |
| 90 | KIAA0522 protein [Homo sapiens] | 3043568 |
| 91 | KIAA0593 protein [Homo sapiens] | 34327962 |
| 92 | KIAA0799 protein [Homo sapiens] | 27529740 |
| 93 | KIAA1064 protein [Homo sapiens] | 20521750 |
| 94 | KIAA1139 protein [Homo sapiens] | 6382016 |
| 95 | KIAA1139 protein [Homo sapiens] | 6382016 |
| 96 | KIAA1619 protein [Homo sapiens] | 10047315 |
| 97 | KIAA1681 protein [Homo sapiens] | 12697907 |
| 98 | KIAA1809 protein [Homo sapiens] | 14017835 |
| 99 | KIAA1856 protein [Homo sapiens] | 14017929 |
| 100 | KIAA1927 protein [Homo sapiens] | 34327988 |
| 101 | matrix metalloproteinase-24 preproprotein [Homo sapiens] | 5729929 |
| 102 | mitogen-activated protein kinase kinase kinase 4 [Homo sapiens] | 55665553 |
| 103 | MSTP054 [Homo sapiens] | 27462066 |
| 104 | myosin X [Homo sapiens] | 7108753 |
| 105 | neuro-endocrine specific protein VGF [Homo sapiens] | 2244659 |
| 106 | neurone-specific enolase [Homo sapiens] | 930063 |
| 107 | neuro-oncological ventral antigen 2 [Homo sapiens] | 119577801 |
| 108 | neurosecretory protein VGF precursor [Homo sapiens] | 17136078 |
| 109 | nucleosome-remodeling factor subunit BPTF isoform 2 [Homo sapiens] | 38788260 |
| 110 | OPN-a [Homo sapiens] | 992948 |
| 111 | osteopontin [Homo sapiens] | 229619843 |
| 112 | osteopontin isoform b precursor [Homo sapiens] | 4759166 |
| 113 | pericentriol material 1 [Homo sapiens] | 450277 |
| 114 | poly-Ig receptor [Homo sapiens] | 514366 |
| 115 | prealbumin [Homo sapiens] | 219978 |
| 116 | proapolipoprotein [Homo sapiens] | 178775 |
| 117 | probable ATP-dependent RNA helicase DDX17 isoform 1 [Homo sapiens] | 38201710 |
| 118 | probable phospholipid-transporting ATPase IM [Homo sapiens] | 50083277 |
| 119 | proline and glutamic acid rich nuclear protein isoform [Homo sapiens] | 3168604 |
| 120 | proline-rich protein 12 [Homo sapiens] | 153792074 |
| 121 | proprotein convertase subtilisin/kexin type 1 inhibitor precursor [Homo sapiens] | 7019519 |
| 122 | prostaglandin D2 synthase 21kDa (brain) [Homo sapiens] | 55962673 |
| 123 | prostaglandin-D synthase (EC 5.3.99.2) - human (fragments) | 2135996 |
| 124 | prostaglandin-H2 D-isomerase [Homo sapiens] | 32171249 |
| 125 | protocadherin 15 [Homo sapiens] | 123221574 |
| 126 | putative protein FAM90A7 [Homo sapiens] | 211971097 |
| 127 | PX serine/threonine kinase isoform 3 [Homo sapiens] | 62184086 |
| 128 | RecName: Full=Alpha-2-HS-glycoprotein; AltName: Full=Ba-alpha-2-glycoprotein; AltName: Full=Alpha-2-Z-globulin; AltName: Full=Fetuin-A;Contains: RecName: Full=Alpha-2-HS-glycoprotein chain A; Contains: RecName: Full=Alpha-2-HS-glycoprotein chain B; | 112910 |
| 129 | RecName: Full=Formin-1; AltName: Full=Limb deformity protein homolog | 158564317 |
| 130 | RecName: Full=Formin-like protein 2; AltName: Full=Formin homology 2 domain-containing protein 2 | 238054383 |
| 131 | RecName: Full=Ladybird homeobox corepressor 1-like protein; AltName: Full=LBX1 corepressor 1-like protein; AltName: Full=Functional Smad-suppressing element on chromosome 18; Short=Fussel-18 | 189082904 |
| 132 | RecName: Full=Mineralocorticoid receptor; Short=MR; AltName: Full=Nuclear receptor subfamily 3 group C member 2 | 126885 |
| 133 | RecName: Full=Secretogranin-1; AltName: Full=Secretogranin I; Short=SgI;AltName: Full=Chromogranin-B; Short=CgB; Contains: RecName: Full=GAWK peptide; Contains: RecName: Full=CCB peptide; Flags: Precursor | 134461 |
| 134 | RING finger protein 44 [Homo sapiens] | 7662486 |
| 135 | RNA polymerase II largest subunit [Homo sapiens] | 825713 |
| 136 | RNA-binding protein 33 [Homo sapiens] | 151301053 |
| 137 | secreted phosphoprotein 1 [Homo sapiens] | 114319017 |
| 138 | serotransferrin precursor [Homo sapiens] | 4557871 |
| 139 | SERPINC1 protein [Homo sapiens] | 18490839 |
| 140 | serum albumin [Homo sapiens] | 62113341 |
| 141 | serum albumin precursor [Homo sapiens] | 6013427 |
| 142 | serum albumin precursor [Homo sapiens] | 6013427 |
| 143 | sodium channel 2 [Homo sapiens] | 1871170 |
| 144 | sparc/osteonectin cwcv and Kazal-like domains proteoglycan precursor variant [Homo sapiens] | 62088982 |
| 145 | talin 2 [Homo sapiens] | 21666571 |
| 146 | talpid3 protein [Homo sapiens] | 154813197 |
| 147 | testican [Homo sapiens] | 3282168 |
| 148 | testican-1 precursor [Homo sapiens] | 4759164 |
| 149 | transcriptional-regulating factor 1 [Homo sapiens] | 15812226 |
| 150 | transferrin [Homo sapiens] | 115394517 |
| 151 | transferrin [Homo sapiens] | 37747855 |
| 152 | transferrin isoform CRA_a [Homo sapiens] | 119599570 |
| 153 | transformation/transcription domain-associated protein isoform CRA_d [Homo sapiens] | 119597102 |
| 154 | translocated to HRX in t(11;19) leukemia [Homo sapiens] | 182110 |
| 155 | transmembrane secretory component [Homo sapiens] | 238236 |
| 156 | TRF1-interacting ankyrin-related ADP-ribose polymerase [Homo sapiens] | 3929219 |
| 157 | tumor necrosis factor ligand superfamily member 6 [Homo sapiens] | 4557329 |
| 158 | ubiquitin carboxyl-terminal hydrolase 51 [Homo sapiens] | 41152235 |
| 159 | unknown [Homo sapiens] | 3924672 |
| 160 | unnamed protein product [Homo sapiens] | 158254894 |
| 161 | unnamed protein product [Homo sapiens] | 158255114 |
| 162 | unnamed protein product [Homo sapiens] | 158258947 |
| 163 | unnamed protein product [Homo sapiens] | 16553207 |
| 164 | unnamed protein product [Homo sapiens] | 194374245 |
| 165 | unnamed protein product [Homo sapiens] | 194383314 |
| 166 | unnamed protein product [Homo sapiens] | 194383506 |
| 167 | unnamed protein product [Homo sapiens] | 194391080 |
| 168 | unnamed protein product [Homo sapiens] | 21749428 |
| 169 | unnamed protein product [Homo sapiens] | 21750524 |
| 170 | unnamed protein product [Homo sapiens] | 21754391 |
| 171 | unnamed protein product [Homo sapiens] | 21756412 |
| 172 | unnamed protein product [Homo sapiens] | 221045134 |
| 173 | unnamed protein product [Homo sapiens] | 28590 |
| 174 | unnamed protein product [Homo sapiens] | 34532827 |
| 175 | unnamed protein product [Homo sapiens] | 34534877 |
| 176 | unnamed protein product [Homo sapiens] | 47077255 |
| 177 | unnamed protein product [Homo sapiens] | 7022113 |
| 178 | unnamed protein product [Homo sapiens] | 7023440 |
| 179 | uromodulin-like protein [Homo sapiens] | 48762454 |
| 180 | voltage-operated calcium channel alpha-1 subunit [Homo sapiens] | 443761 |
| 181 | Wiskott-Aldrich syndrome protein [Homo sapiens] | 4507909 |
| 182 | Wiskott-Aldrich syndrome-like [Homo sapiens] | 51095090 |
| 183 | ZFHX4 protein [Homo sapiens] | 34783722 |
| 184 | zinc finger CCHC domain-containing protein 2 [Homo sapiens] | 170016069 |
| 185 | zinc finger UBR1 type 1 isoform CRA_d [Homo sapiens] | 119615273 |
| 186 | zinc-finger homeodomain protein 4 [Homo sapiens] | 109638254 |
