# Supplementary material for: Monitoring CSF Proteome Alterations in Amyotrophic Lateral Sclerosis: Obstacles and Perspectives in Translating a Novel Marker Panel to the Clinic
Source: PLoS One. 2012 Sep 6;7(9):e44401. doi: 10.1371/journal.pone.0044401 (PMC3435306; doi:10.1371/journal.pone.0044401)
Supplement: Table S2 — Comparison between MRM, MS/MS and BisoGenet pathway. (PDF) [file pone.0044401.s004.pdf]

Table S2
Comparison between MRM, MS/MS and BisoGenet pathway

**Multiple Reaction Monitoring Quantification**				**MS/MS**	**Pathway Analysis**	
NextGen ID	FC (ALS/control)	Gene Symbol	Protein Name	Identification	Gene Symbol	Protein Name
ORM1	1,06	A1AG1_HUMAN	Alpha-1-acid glycoprotein 1			
SERPINA3	1,08	AACT_HUMAN	Alpha-1-antichymotrypsin			
SERPINA1	0,96	A1AT_HUMAN	Alpha-1-antitrypsin ❶	◊	A1AT	Alpha-1-antitrypsin ❶
A1BG	0,97	A1BG_HUMAN	Alpha-1B-glycoprotein		ABCF3	ATP-binding cassette, sub-family F (GCN20), member 3
AHSG	0,74	FETUA_HUMAN	Alpha-2-HS-glycoprotein	*	ACP5	Acid phosphatase 5, tartrate resistant
APP	0,97	A4_HUMAN	Amyloid beta A4 protein		ADAMTS4	ADAM metallopeptidase with Thrombospondin type 1 motif, 4
APLP2	1,03	APLP2_HUMAN	Amyloid-like protein 2		ATXN1	Ataxin 1
AGT	1,04	ANGT_HUMAN	Angiotensinogen		CSNK2A1	Casein kinase 2, alpha 1 polypeptide
APOA1	0,98	APOA1_HUMAN	Apolipoprotein A-I		CSNK2A1P	Casein kinase 2, alpha 1 polypeptide pseudogene
APOA4	0,80	APOA4_HUMAN	Apolipoprotein A-IV		CSNK2A2	Casein kinase 2, alpha prime polypeptide
APOD	1,06	APOD_HUMAN	Apolipoprotein D	*		
APOE	0,95	APOE_HUMAN	Apolipoprotein E	*	CANX	Calnexin
ATRN	1,00	ATRN_HUMAN	Attractin		CASP3	Caspase 3
APOH	0,70	APOH_HUMAN	Beta-2-glycoprotein 1	*	CASP8	Caspase 8
B2M	1,07	B2MG_HUMAN	Beta-2-microglobulin	*	CD44	CD44 antigen
CNDP1	1,04	CNDP1_HUMAN	Beta-Ala-His dipeptidase		CELA1	Chymotrypsin-like elastase 1
CDH13	0,92	CAD13_HUMAN	Cadherin-13		CTSL1	Cathepsin L1
CLSTN1	0,98	CSTN1_HUMAN	Calsyntenin-1		CTSB	Cathepsin B
CTSD	0,91	CATD_HUMAN	Cathepsin D ❷		CTSD	Cathepsin D ❷
CP	0,91	CERU_HUMAN	Ceruloplasmin		CTSG	Cathepsin G
CHI3L1	1,31	CH3L1_HUMAN	Chitinase-3-like protein 1		CTSS	Cathepsin S
CHGA	0,90	CMGA_HUMAN	Chromogranin-A ❶		CHGA	Chromogranin-A ❶
CLU	0,91	CLUS_HUMAN	Clusterin			
C2	0,94	CO2_HUMAN	Complement C2			
C3	0,94	CO3_HUMAN	Complement C3 ❸		C4A	Complement component 4A ❸
C8B	0,96	CO8B_HUMAN	Complement component C8 beta chain			
C9	0,92	CO9_HUMAN	Complement component C9			
CFH	0,96	CFAH_HUMAN	Complement factor H			
CFHR2	0,59	FHR2_HUMAN	Complement factor H-related protein 2			
CNTN1	1,01	CNTN1_HUMAN	Contactin-1			
CNTN2	0,99	CNTN2_HUMAN	Contactin-2			
SERPINA6	1,13	CBG_HUMAN	Corticosteroid-binding globulin			
CST3	0,96	CYTC_HUMAN	Cystatin-C ❶	◊	CST3	Cystatin C ❶
ENPP2	0,95	ENPP2_HUMAN	Ectonucleotide pyrophosphatase/phosphodiesterase family member 2		CSTB	Cystatin B (stefin B)
EXTL2	1,01	EXTL2_HUMAN	Exostosin-like 2		CTNNBL	Catenin, beta like 1
FN1	0,97	FINC_HUMAN	Fibronectin		CTRB1	Chymotrypsinogen B1
GSN	0,87	GELS_HUMAN	Gelsolin		DERL2	Der1-like domain family, member 2
HPX	0,83	HEMO_HUMAN	Hemopexin		DERL3	Der1-like domain family, member 3
SERPIND1	0,88	HEP2_HUMAN	Heparin cofactor 2		ELANE	Elastase, neutrophil expressed
HABP2	0,81	HABP2_HUMAN	Hyaluronan-binding protein 2			
IGHA1	0,85	IGHA1_HUMAN	Ig alpha-1 chain C region		GRB2	Growth factor receptor-bound protein 2
IGHM	0,97	IGHM_HUMAN	Ig mu chain C region			
IGFBP3	0,67	IBP3_HUMAN	Insulin-like growth factor-binding protein 3 ❸		IGFBP5	Insulin-like growth factor binding protein 5 ❸
Bri2-23	0,65	ITM2B_HUMAN	Integral membrane protein 2B (23AA peptide released from ITM2B (BRI2))		KLK3	Kallikrein-related peptidase 3
KLK6	1,03	KLK6_HUMAN	Kallikrein-6 ❸		KLK5	Kallikrein-related peptidase 5 ❸
KNG1	0,86	KNG1_HUMAN	Kininogen-1		KLK13	Kallikrein-related peptidase 13
LRG1	1,05	A2GL_HUMAN	Leucine-rich alpha-2-glycoprotein		ITGA5	Integrin, alpha 5 (fibronectin receptor, alpha polypeptide)
LUM	0,93	LUM_HUMAN	Lumican		ITGA9	Integrin, alpha 9
B3GNT1	0,94	B3GN1_HUMAN	N-acetyllactosaminide beta-1,3-N-acetylglucosaminyltransferase		ITGAV	Integrin, alpha V (vitronectin receptor, alpha polypeptide, antigen CD51)
PGLYRP2	0,89	PGRP2_HUMAN	N-acetylmuramoyl-L-alanine amidase		ITGB1	Integrin, beta 1 (fibronectin receptor, beta polypeptide, antigen CD29 includes MDF2, MSK12)
NCAM1	1,02	NCAM1_HUMAN	Neural cell adhesion molecule 1		MMP11	Matrix metallopeptidase 11 (stromelysin 3)
NRCAM	1,01	NRCAM_HUMAN	Neuronal cell adhesion molecule		MMP26	Matrix metallopeptidase 26
NPTXR	0,97	NPTXR_HUMAN	Neuronal pentraxin receptor		MMP3	Matrix metallopeptidase 3 (stromelysin 1, progelatinase)

NPTX1	1,00	NPTX1_HUMAN	Neuronal pentraxin-1			MMP7	Matrix metallopeptidase 7 (matrilysin, uterine)
VGF	0,79	VGF_HUMAN	Neurosecretory protein VGF ❶		◊	VGF	Neurosecretory protein VGF ❶
SERPINI1	0,95	NEUS_HUMAN	Neuroserpin			LRP1	Low density lipoprotein receptor-related protein 1
SPP1	0,94	OSTP_HUMAN	Secreted phosphoprotein 1 ❶		◊	SPP1	Secreted phosphoprotein 1 ❶
SERPINF1	0,91	PEDF_HUMAN	Pigment epithelium-derived factor			PDLIM7	PDZ and LIM domain 7 (enigma)
SERPING1	0,92	IC1_HUMAN	Plasma protease C1 inhibitor			PDZD2	PDZ domain containing 2
PLG	0,89	PLMN_HUMAN	Plasminogen ❷			PLG	Plasminogen ❷
PCSK1N	0,94	PCSK1_HUMAN	ProSAAS	*		PRSS1	Protease, serine, 1 (trypsin 1)
PTGDS	1,01	PTGDS_HUMAN	Prostaglandin-H2 D-isomerase	*		PRSS2	Protease, serine, 2 (trypsin 2)
AMBP	0,72	AMBP_HUMAN	Protein AMBP			PRSS3	Protease, serine, 3
CUTA	1,04	CUTA_HUMAN	Protein CutA			PRTN3	Proteinase 3
PARK7	0,87	PARK7_HUMAN	Protein DJ-1			PRKG1	Protein kinase, cGMP-dependent, type I
NELL2	0,98	NELL2_HUMAN	Protein kinase C-binding protein NELL2 ❸			PRKCA	Protein kinase C, alpha ❸
F2	0,79	THRB_HUMAN	Thrombin ❷			F2	Thrombin ❷
RBP4	0,81	RET4_HUMAN	Retinol-binding protein 4				
CHGB	1,03	SCG1_HUMAN	Secretogranin-1	*			
SCG2	0,92	SCG2_HUMAN	Secretogranin-2				
SCG3	0,93	SCG3_HUMAN	Secretogranin-3 ❷			SCG3	Secretogranin-3 ❷
SEPP1	0,78	SEPP1_HUMAN	Selenoprotein P				
TF	0,93	TRFE_HUMAN	Serotransferrin	*			
PON1	1,10	PON1_HUMAN	Serum paraoxonase/arylesterase 1			RAP2A	RAP2A, member of RAS oncogene family
SPARCL1	0,87	SPRL1_HUMAN	SPARC-like protein 1	*		SGTA	Small glutamine-rich tetratricopeptide repeat (TPR)-containing, alpha
CLEC3B	0,83	TETN_HUMAN	Tetranectin				
TTR	0,95	TTHY_HUMAN	Transthyretin	*		SSR1	Signal sequence receptor, alpha
GC	0,94	VTDB_HUMAN	Vitamin D-binding protein			UBC	Ubiquitin C
PROS1	0,90	PROS_HUMAN	Vitamin K-dependent protein S			UBQLN4	Ubiquilin 4
AZGP1	0,91	ZA2G_HUMAN	Zinc-alpha-2-glycoprotein			UGGT1	UDP-glucose glycoprotein glucosyltransferase 1

❶ MRM assay matched classifier protein
❷ additional match between MRM assay and pathway
❸ closely related proteins in MRM panel and pathway
◊ MS/MS independently identified classifier protein
* MS/MS independently identified protein
Fold change: >1: upregulated in ALS CSF samples; <1: downregulated in ALS CSF samples
