# Supplementary material for: Monitoring CSF Proteome Alterations in Amyotrophic Lateral Sclerosis: Obstacles and Perspectives in Translating a Novel Marker Panel to the Clinic
Source: PLoS One. 2012 Sep 6;7(9):e44401. doi: 10.1371/journal.pone.0044401 (PMC3435306; doi:10.1371/journal.pone.0044401)
Supplement: Table S3 — DAVID analysis of the 59 proteins from the BisoGenet pathway. (DOC) [file pone.0044401.s005.doc]

**Table S3**

**DAVID Analysis of the 59 Proteins from the BisoGenet pathway**

**S3a**

Enriched UP Feature Expression

| **Category** | **Term** | **Proteins** | **%** | **P-Value** | **BH corr.** |
| --- | --- | --- | --- | --- | --- |
| UP_SEQ_FEATURE | signal peptide | 38 | 66,7 | 3,6E-16 | 3,9E-14 |
| UP_SEQ_FEATURE | propeptide:Activation peptide | 13 | 22,8 | 1,8E-18 | 6,3E-16 |
| UP_SEQ_FEATURE | domain:Peptidase S1 | 13 | 22,8 | 2,0E-16 | 3,9E-14 |
| UP_SEQ_FEATURE | disulfide bond | 33 | 57,9 | 2,4E-13 | 2,1E-11 |
| UP_SEQ_FEATURE | active site:Charge relay system | 13 | 22,8 | 3,6E-13 | 2,5E-11 |
| UP_SEQ_FEATURE | sequence variant | 46 | 80,7 | 3,5E-3 | 6,6E-2 |
| UP_SEQ_FEATURE | glycosylation site:N-linked (GlcNAc...) | 27 | 47,4 | 3,1E-5 | 1,1E-3 |
| UP_SEQ_FEATURE | mutagenesis site | 11 | 19,3 | 7,1E-2 | 5,9E-1 |
| UP_SEQ_FEATURE | metal ion-binding site:Calcium; via carbonyl oxygen | 6 | 10,5 | 7,3E-8 | 4,2E-6 |
| UP_SEQ_FEATURE | metal ion-binding site:Calcium | 6 | 10,5 | 2,5E-6 | 1,2E-4 |
| UP_SEQ_FEATURE | short sequence motif:Cysteine switch | 5 | 8,8 | 7,5E-6 | 3,3E-4 |
| UP_SEQ_FEATURE | metal ion-binding site:Calcium 2; via carbonyl oxygen | 4 | 7,0 | 2,0E-4 | 6,4E-3 |
| UP_SEQ_FEATURE | metal ion-binding site:Calcium 1 | 4 | 7,0 | 3,1E-4 | 9,0E-3 |
| UP_SEQ_FEATURE | metal ion-binding site:Calcium 2 | 4 | 7,0 | 5,6E-4 | 1,4E-2 |
| UP_SEQ_FEATURE | site:Required for specificity | 3 | 5,3 | 2,5E-5 | 9,8E-4 |
| UP_SEQ_FEATURE | metal ion-binding site:Zinc 2; in inhibited form | 3 | 5,3 | 3,7E-4 | 1,0E-2 |
| UP_SEQ_FEATURE | metal ion-binding site:Zinc 2; catalytic | 3 | 5,3 | 7,5E-4 | 1,7E-2 |
| UP_SEQ_FEATURE | metal ion-binding site:Calcium 3; via carbonyl oxygen | 3 | 5,3 | 1,7E-3 | 3,7E-2 |
| UP_SEQ_FEATURE | metal ion-binding site:Calcium 3 | 3 | 5,3 | 3,5E-3 | 6,9E-2 |
| UP_SEQ_FEATURE | metal ion-binding site:Calcium 1; via carbonyl oxygen | 3 | 5,3 | 4,0E-3 | 7,0E-2 |
| UP_SEQ_FEATURE | metal ion-binding site:Zinc 1 | 3 | 5,3 | 2,0E-2 | 3,0E-1 |
| UP_SEQ_FEATURE | glycosylation site:O-linked (GalNAc...) | 3 | 5,3 | 3,0E-2 | 3,7E-1 |
| UP_SEQ_FEATURE | domain:Kringle 1 | 2 | 3,5 | 2,0E-2 | 2,9E-1 |
| UP_SEQ_FEATURE | domain:Kringle 2 | 2 | 3,5 | 2,0E-2 | 2,9E-1 |
| UP_SEQ_FEATURE | site:Reactive site | 2 | 3,5 | 2,6E-2 | 3,4E-1 |
| UP_SEQ_FEATURE | short sequence motif:Secondary area of contact | 2 | 3,5 | 3,5E-2 | 4,0E-1 |
| UP_SEQ_FEATURE | short sequence motif:GFFKR motif | 2 | 3,5 | 4,3E-2 | 4,6E-1 |
| UP_SEQ_FEATURE | repeat:FG-GAP 7 | 2 | 3,5 | 5,4E-2 | 5,3E-1 |
| UP_SEQ_FEATURE | repeat:FG-GAP 5 | 2 | 3,5 | 5,4E-2 | 5,3E-1 |
| UP_SEQ_FEATURE | repeat:FG-GAP 4 | 2 | 3,5 | 5,4E-2 | 5,3E-1 |
| UP_SEQ_FEATURE | repeat:FG-GAP 1 | 2 | 3,5 | 5,4E-2 | 5,3E-1 |
| UP_SEQ_FEATURE | repeat:FG-GAP 2 | 2 | 3,5 | 5,4E-2 | 5,3E-1 |
| UP_SEQ_FEATURE | repeat:FG-GAP 6 | 2 | 3,5 | 5,4E-2 | 5,3E-1 |
| UP_SEQ_FEATURE | repeat:FG-GAP 3 | 2 | 3,5 | 5,4E-2 | 5,3E-1 |
| UP_SEQ_FEATURE | domain:Hemopexin-like 4 | 2 | 3,5 | 6,3E-2 | 5,7E-1 |
| UP_SEQ_FEATURE | domain:Hemopexin-like 3 | 2 | 3,5 | 6,3E-2 | 5,7E-1 |
| UP_SEQ_FEATURE | domain:Hemopexin-like 1 | 2 | 3,5 | 6,5E-2 | 5,7E-1 |
| UP_SEQ_FEATURE | domain:Hemopexin-like 2 | 2 | 3,5 | 6,5E-2 | 5,7E-1 |
| UP_SEQ_FEATURE | metal ion-binding site:Zinc; in inhibited form | 2 | 3,5 | 9,2E-2 | 6,8E-1 |

**S3b**

**Enriched GO Cellular Components**

| **Category** | **Term** | **Proteins** | **%** | **P-Value** | **BH corr.** |
| --- | --- | --- | --- | --- | --- |
| GOTERM_CC_FAT | extracellular region | 30 | 52,6 | 3,6E-11 | 4,8E-9 |
| GOTERM_CC_FAT | extracellular region part | 20 | 35,1 | 2,6E-9 | 1,7E-7 |
| GOTERM_CC_FAT | extracellular space | 17 | 29,8 | 6,6E-9 | 3,0E-7 |
| GOTERM_CC_FAT | membrane-bounded vesicle | 10 | 17,5 | 4,2E-4 | 1,4E-2 |
| GOTERM_CC_FAT | extracellular matrix | 8 | 14,0 | 4,6E-4 | 1,2E-2 |
| GOTERM_CC_FAT | vesicle | 10 | 17,5 | 1,4E-3 | 3,0E-2 |
| GOTERM_CC_FAT | cell surface | 7 | 12,3 | 2,8E-3 | 5,2E-2 |
| GOTERM_CC_FAT | pigment granule | 4 | 7,0 | 5,6E-3 | 9,1E-2 |
| GOTERM_CC_FAT | melanosome | 4 | 7,0 | 5,6E-3 | 9,1E-2 |
| GOTERM_CC_FAT | integrin complex | 3 | 5,3 | 6,1E-3 | 8,8E-2 |
| GOTERM_CC_FAT | cytoplasmic membrane-bounded vesicle | 8 | 14,0 | 6,5E-3 | 8,5E-2 |
| GOTERM_CC_FAT | proteinaceous extracellular matrix | 6 | 10,5 | 9,5E-3 | 1,1E-1 |
| GOTERM_CC_FAT | lysosome | 5 | 8,8 | 1,1E-2 | 1,1E-1 |
| GOTERM_CC_FAT | lytic vacuole | 5 | 8,8 | 1,1E-2 | 1,1E-1 |
| GOTERM_CC_FAT | cytoplasmic vesicle | 8 | 14,0 | 1,5E-2 | 1,4E-1 |
| GOTERM_CC_FAT | vacuole | 5 | 8,8 | 1,9E-2 | 1,7E-1 |
| GOTERM_CC_FAT | ruffle | 3 | 5,3 | 3,0E-2 | 2,4E-1 |
| GOTERM_CC_FAT | external side of plasma membrane | 4 | 7,0 | 3,2E-2 | 2,4E-1 |
| GOTERM_CC_FAT | synaptosome | 3 | 5,3 | 4,7E-2 | 3,2E-1 |
| GOTERM_CC_FAT | ruffle membrane | 2 | 3,5 | 4,8E-2 | 3,1E-1 |
| GOTERM_CC_FAT | focal adhesion | 3 | 5,3 | 6,5E-2 | 3,8E-1 |
| GOTERM_CC_FAT | cell-substrate adherens junction | 3 | 5,3 | 6,9E-2 | 3,8E-1 |
| GOTERM_CC_FAT | cell fraction | 9 | 15,8 | 7,0E-2 | 3,7E-1 |
| GOTERM_CC_FAT | cell-substrate junction | 3 | 5,3 | 7,6E-2 | 3,8E-1 |
| GOTERM_CC_FAT | receptor complex | 3 | 5,3 | 8,1E-2 | 3,9E-1 |
| GOTERM_CC_FAT | leading edge membrane | 2 | 3,5 | 9,0E-2 | 4,1E-1 |

**S3c**

**Enriched KEGG Pathways**

| **Category** | **Term** | **Proteins** | **%** | **P-Value** | **BH corr.** |
| --- | --- | --- | --- | --- | --- |
| KEGG_PATHWAY | Neuroactive ligand-receptor interaction | 7 | 12,3 | 3,9E-3 | 8,4E-2 |
| KEGG_PATHWAY | Lysosome | 6 | 10,5 | 6,2E-4 | 4,1E-2 |
| KEGG_PATHWAY | Focal adhesion | 6 | 10,5 | 6,7E-3 | 1,1E-1 |
| KEGG_PATHWAY | Pathways in cancer | 6 | 10,5 | 4,6E-2 | 3,6E-1 |
| KEGG_PATHWAY | ECM-receptor interaction | 5 | 8,8 | 1,6E-3 | 5,1E-2 |
| KEGG_PATHWAY | Complement and coagulation cascades | 4 | 7,0 | 8,2E-3 | 1,0E-1 |
| KEGG_PATHWAY | Antigen processing and presentation | 4 | 7,0 | 1,4E-2 | 1,4E-1 |
| KEGG_PATHWAY | Wnt signaling pathway | 4 | 7,0 | 6,3E-2 | 4,2E-1 |
| KEGG_PATHWAY | Arrhythmogenic right ventricular cardiomyopathy (ARVC) | 3 | 5,3 | 7,8E-2 | 4,5E-1 |
| KEGG_PATHWAY | Hypertrophic cardiomyopathy (HCM) | 3 | 5,3 | 9,4E-2 | 4,8E-1 |
